# Wine Lees as Source of Antioxidant Molecules: Green Extraction Procedure and Biological Activity

**DOI:** 10.3390/antiox12030622

**Published:** 2023-03-02

**Authors:** Michele De Luca, Donatella Restuccia, Umile Gianfranco Spizzirri, Pasquale Crupi, Giuseppina Ioele, Beatrice Gorelli, Maria Lisa Clodoveo, Simona Saponara, Francesca Aiello

**Affiliations:** 1 Department of Pharmacy, Health and Nutritional Sciences, University of Calabria, 87036 Rende, Italy; 2 Interdisciplinary Department of Medicine, University of Bari “Aldo Moro”, 70124 Bari, Italy; 3 Department of Life Sciences, University of Siena, Via Aldo Moro 2, 53100 Siena, Italy

**Keywords:** wine lees, green extraction procedure, principal component analysis, partial least square regression, antioxidant properties

## Abstract

An ultrasound-assisted extraction method, employing ethanol and water as solvents at low temperature (30 °C) and reduced time (15 min), was proposed to extract bioactive molecules from different cultivars (*Magliocco Canino*, *Magliocco Rosato*, *Gaglioppo*, and *Nocera Rosso*) of wine lees. All the extract yields were evaluated and their contents of phenolic acids, flavonoids, and total polyphenols were determined by means of colorimetric assays and high-performance liquid chromatography coupled with diode-array detection (HPLC-DAD) and Fourier transform infrared (FTIR) techniques. Radical scavenging assays were performed and the *Magliocco Canino* extracted with a hydroalcoholic mixture returned the best results both against ABTS (0.451 mg mL^−1^) and DPPH (0.395 mg mL^−1^) radicals. The chemometric algorithms principal component analysis (PCA) and partial least square regression (PLS) were used to process the data obtained from all qualitative–quantitative sample determinations with the aim of highlighting data patterns and finding possible correlations between composition and antioxidant features of the different wine lees cultivars and the extraction procedures. Wine lees from *Magliocco Canino* and *Magliocco Rosato* were found to be the best vegetable matrices in terms of metabolite content and antioxidant properties. The components extracted with alcoholic or hydroalcoholic solvents, specifically (−)-epigallocatechin gallate, chlorogenic acid, and *trans*-caftaric acid, were found to be correlated with the antioxidant capacity of the extracts. Multivariate data processing was able to identify the compounds related to the antioxidant features. Two PLS models were optimized by using their concentration levels to predict the IC_50_ values of the extracts in terms of DPPH and ABTS with high values of correlation coefficient R^2^, 0.932 and 0.824, respectively, and a prediction error lower than 0.07. Finally, cellular (SH-SY5Y cells) antioxidant assays were performed on the best extract (the hydroalcoholic extract of *Magliocco Canino cv*) to confirm its biological performance against radical species. All these recorded data strongly outline the aptness of valorizing wine lees as a valuable source of antioxidants.

## 1. Introduction

Wine is produced in all parts of the world, but France, Italy, Spain, and some emerging countries such as Australia, Chile, South Africa, and the United States represent the main world producers [1]. The market data highlight the extreme complexity of the wine sector, often based on thousands of small farms that produce small quantities of wine, together with other large companies with high wine production [2]. Italy, together with France, ranks at the top of the classification of wine-producing countries. Italy is still the leading wine-producing country with 44,500 hectoliters, while European Union countries reached 145,000 hectoliters in 2021, with a substantial decrease of 13% (−21,000 hectoliters) compared to 2020, almost achieving 60% of world wine production [3].

However, the wine industry generates enormous quantities of waste every year, among which are pomace, grape seeds, stems, leaves, lees, and cellar waste. The literature data report that for each produced hectoliter of wine, 20 kg of pomace, 3.85 kg of stalks, and 6.36 kg of lees and clarification solids are produced [4]. Their disposal, even in the face of the considerable quantities and their molecular composition, has an environmental impact that should not be underestimated. For this reason, it is important to adopt strategies for the recovery and valorization of wine wastes. The reduction in by-products and waste produced during the winemaking process plays a crucial role in providing a green and clean vision of this agri-food sector. The aim can be easily realized by providing new and innovative strategies able to transform the winemaking waste into high-added-value products. Winemaking wastes represent a valuable supply of active molecules, mainly polyphenols, that can be largely reused as supplements in pharmaceutical and biomedical fields, as well as in the food industry. Recently, mixtures of grape seeds and skins were used to produce extracts useful as food supplements [5,6]. Nevertheless, wine lees generated in post-fermentation are poorly investigated, even though the lees represent about 14% of the total organic waste produced during winemaking [7].

The lees are the residue accumulated after the fermentation of the wine consisting predominantly of yeasts and impurities originating from grapes. Two main fractions comprise lees by-products [8]: a combination of proteins, insoluble carbohydrates, organic acids, yeasts, inorganic salts, and phenolic molecules provides the solid fraction [9], while ethanol, acetic acid, and lactic acid are the main compounds of the liquid fraction [10]. Environmental conditions, grape variety, and regions of origin are agronomic parameters that deeply influence the chemical composition of lees [11]. Lees have usually been a key raw material to produce ethanol and tartaric acid for application in the food industry [12] and represent a resource of high-added-value substances with remarkable antioxidant capacity and the ability to prevent cardiovascular diseases, as well as a remarkable mix of anti-inflammatory and anticancer molecules [13].

Starting from lees produced by winemaking with different grape cultivars, the challenge of this research was to propose an innovative and eco-friendly extraction protocol able to provide a mix of chemical molecules with notable antioxidant features. In this regard, different solvents were tested, while ultrasound-assisted extraction was employed to reduce the time and temperature of the process, preserving the chemical and biological integrity of the active compounds. The extracts were deeply characterized by chromatographic and spectroscopic methodologies and antioxidant performance and the collected data were analyzed by chemometric tools in order to highlight data patterns and the relationship between the chemical composition of the samples and their antioxidant capacities.

## 2. Materials and Methods

### 2.1. Chemicals

Gallic acid, (+)-hydrated catechin, (−)-epigallocatechin, procyanidin B_2_, epigallocatechin gallate, (−)-epicatechin, chlorogenic acid, caffeic acid, p-coumaric acid, ferulic acid, Folin–Ciocalteu reagent, sodium carbonate (Na_2_CO_3_) radical 2,2′-diphenyl-1-picrylyhydrazyl (DPPH), radical 2,2′-azino-bis(3-ethylbenzothiazolin-6-sulfonic) (ABTS), sodium nitrite (NaNO_2_), sodium phosphate (Na_3_PO_4_), aluminum chloride (AlCl_3_), hydrochloric acid (HCl), sodium hydroxide (NaOH), absolute ethanol, Dulbecco’s modified Eagle’s medium (DMEM), DMEM without phenol red, phosphate buffer solution (PBS), 2′,7′-dichlorofluorescein diacetate (DCF-DA), hydrogen peroxide (H_2_O_2_), *L*-glutamine, penicillin, streptomycin, and Whatman No. 3 filter paper were purchased from Sigma Aldrich (Sigma Chemical Co., St. Louis, MO, USA). Delphinidin-3-*O*-glucoside (DG), cyanidin-3*O*-glucoside (CG), petunidin-3*O*-glucoside (PTG), peonidin-3*O*-glucoside (POG), and malvidin-3*O*-glucoside (MG) were purchased from Extrasynthese (Genay, France). HPLC-grade water and acetonitrile were purchased from VWR (Chromasolv, VWR International Srl, Milano, Italy). 

### 2.2. Sample Collection and Preparation

A total of four lees samples of autochthonous Calabria *Vitis vinifera* red grape varieties (*Nocera Rosso* (1), *Magliocco Rosato* (2), *Magliocco Canino* (3), and *Gaglioppo* (4)) were collected in September 2021 from a local producer (Azienda Agricola Donna Fidelia, Belvedere Marittimo, Italy). The grapes were harvested at technological maturation, and the agronomic practices as well as the winemaking process were the same for all analyzed samples. The removal of the ethanol from the lees samples was accomplished by rotary evaporation (ALC Multispeed Centrifuge Thermo Electron Corporation, Waltham, MA, USA ) for 15 min at 35 °C and the concentrated solution was frozen at −18 °C and then freeze-dried at a pressure of 0.45 mbar (Micro Modulyo, Edwards). Freeze-dried lees samples were crushed and kept in hermetically sealed containers at −18 °C until further analysis.

### 2.3. Wine Lees Extraction

Lees samples were extracted as described by Carullo et al. [13]. Two hundred mL of ethanol (E), HCl solution at pH = 2.0 (W), or hydroalcoholic solution (50% *v*/*v*) acidified at pH 2.0 with HCl (WE) were added to 1.0 g of a freeze-dried sample of each cultivar, and the samples were extracted using an ultrasound-bath Branson model 3800-CPXH (Milan, Italy) at 30 °C (10 cycles/sec) for 15 min at an ultrasonic frequency of 40 kHz. The supernatants were decanted and the solid was removed, while the solution was evaporated, frozen, and freeze-dried to provide a vaporized solid. Wine lees and sample preparation were performed in triplicate.

### 2.4. Chemical Characterization of Lyophilized Wine Lees

#### 2.4.1. HPLC-DAD Analysis of Lyophilized Wine Lees 

High-performance liquid chromatography coupled to diode array detection (HPLC-DAD) analysis of polyphenols was performed by an HPLC 1260 system equipped with a degasser, quaternary pump solvent delivery, thermostatic column compartment, autosampler, and diode array detector (DAD) in a series configuration (Agilent Technologies, Palo Alto, CA, USA). The wine lees extracts were resuspended in 1.5 mL ethanol/water (70:30, *v*/*v*), filtered through 0.45 μm pore size regenerated cellulose filters (VWR International Srl, Milano, Italy), and injected onto a reversed stationary phase column, ZORBAX Eclipse Plus C_18_ (250 × 4.6 mm i.d., particle size 5 μm, Agilent Technologies, Palo Alto, CA, USA) protected by a C_18_ Guard Cartridge (4.0 × 2.0 mm i.d., Phenomenex, Torrance, CA, USA). HPLC separation was accomplished by using a binary mobile phase composed of (solvent A) H_2_O/formic acid 10% (*v*/*v*) and (solvent B) acetonitrile. The following gradient was employed: 0 min, 5% B; 10 min, 13% B; 20 min, 15% B; 30 min, 22% B; 50 min, 22% B; 51 min, 100% B; 61 min, 100% B; and 62 min, 5% B. This was followed by washing and re-equilibrating the column. The column temperature was controlled at 25 °C, and the flow was maintained at 0.7 mL min^−1^. DAD wavelengths were set at 280, 320, 360, and 520 nm corresponding to the maximum absorptions of flavan-3-ols and benzoic acids, hydroxycinnamic acids, flavonols, and anthocyanins, respectively.

The tentative identification of the compounds in the extracts was achieved by matching the positions of absorption maxima (λ_max_), absorption spectra profiles, and retention times (RT) with those from commercially available pure standards and studies already reported in the literature for comparable matrices [14,15]. Moreover, the quantification was performed using the calibration curves in the concentration range of 100–0.2 µg mL^−1^ of malvidin-3*O*-glucoside (R^2^ = 0.9983), caffeic acid (R^2^ = 0.9988), epicatechin (R^2^ = 0.9944), and gallic acid (R^2^ = 0.9991).

#### 2.4.2. Fourier Transform Infrared Analysis of Lyophilized Wine Lees

The infrared fingerprints of the lyophilized wine lees samples were acquired by using the Spectrum Two infrared spectrometer (Perkin Elmer Italia Spa, Milan, Italy) supplied with an attenuated total reflection (ATR) accessory with a ZnSe crystal on the sampling flat plate. The ATR detector was cleaned with ethanol after each spectral collection and then dried. The infrared spectrum of the laboratory air was considered as background to check the instrumental status and H_2_O/CO_2_ interferences. The FTIR spectra of the samples (3 samples for each extraction procedure), placed on the ATR detector, were recorded in triplicate in the range 4000–450 cm^−1^, with 32 scans and a resolution of 4 cm^−1^. The fingerprint analysis of samples was focused on a specific wavenumber range, the variables selection was carried out by taking out the spectral window 4000–1850 cm^−1^ [16,17]. The interval 4000–2500 cm^−1^ contained mainly signals of the stretching vibrations due to -OH functions and aromatic bonds (C-H), and information about phenolic compounds was relatively poor. The 2500–1850 cm^−1^ interval also contained no relevant spectral information besides the band corresponding to CO_2_. The remaining 1800–450 cm^−1^ interval was set in matrix form and subjected to chemometric modeling after opportune pre-treatment and signal transformation from reflectance to absorbance data.

#### 2.4.3. Polyphenols Total Content

The total phenol content (TPC) of wine lees extracts was determined as described by Spizzirri et al. [18]. In a volumetric flask, 6.0 mL of an aqueous solution of each lees extract, 1.0 mL of Folin–Ciocalteu reagent, and 2.0 mL of Na_2_CO_3_ solution (2% *w*/*v*) were added. After two hours at room temperature, the absorbance was spectrophotometrically measured at 720 nm by a Jasco V-530 UV/Vis spectrometer (Jasco, Tokyo, Japan) against a control. This analysis was performed in triplicate and the TPC value was determined by a standard curve constructed using gallic acid (GA) in the range of 8–40 μM (R^2^ = 0.9988). The TPC for each extract was expressed as mg GA equivalents per gram of dry matrix (mg GA/g).

#### 2.4.4. Phenolic Acid Content

The phenolic acid content (PAC) of each dry matrix sample was evaluated by a literature procedure with some changes [19]. In a volumetric flask (10.0 mL) 1.0 mL of an aqueous solution of each extract, 1.0 mL of HCl (0.5 mol L^−1^), 1.0 mL of Arnov’s reagent (Na_2_MoO_4_ and NaNO_3_ 0.1 mg mL^−1^), 1.0 mL of NaOH (1.0 mol L^−1^), and distilled water were added. The absorbance of the solutions was spectrophotometrically determined at 490 nm. This analysis was performed in triplicate and the PAC value was determined by a standard curve constructed using GA in the range of 10–80 μM (R^2^ = 0.9973). The PAC for each extract was expressed as mg GA equivalents per gram of dry matrix (mg GA/g).

#### 2.4.5. Flavonoid Content

Flavonoid content (FC) in the lees extracts was spectrophotometrically determined by a literature procedure with some changes and expressing the results as the mass of catechin (CT) per gram of dry sample (mg CT g^−1^) [20]. Briefly, in a 5.0 mL volumetric flask, 0.5 mL of an aqueous solution of each extract was added to 0.15 mL of NaNO_2_ (5% *w*/*v*). After 6 min, 0.3 mL of AlCl_3_ (6% *w*/*v*), and after 5 min, 1.0 mL of NaOH 1.0 M were added to the mixture by measuring the absorbance at 510 nm against a control. A calibration curve of CT was built in the concentration range of 10.0–100.0 μM (R^2^ = 0.9975). 

### 2.5. Antioxidant Properties

The antioxidant features of the lees extracts were evaluated by specific measurements able to quantify their scavenger activities in organic and aqueous media against hydrophilic and lipophilic radical species, respectively. 

The scavenger capacity of the extracts in an organic environment was evaluated by analyzing the concentration decrease in DPPH radical, slightly modifying a literature procedure [21]. Briefly, in a volumetric flask (10 mL) 1.0 mL of hydroalcoholic solutions (50:50 *v*/*v*) of each extract, 4.0 mL of a hydroalcoholic mixture (50:50 *v*/*v*), and 5.0 mL of a 200 μM ethanolic solution of DPPH were mixed. The mixture was kept at room temperature for 30 min and the residual concentration of the radical species was spectrophotometrically measured at 517 nm. The scavenging capacity of each lees extract against DPPH was expressed in terms of IC_50_. Ascorbic acid was used as a positive control.

The scavenging activity of the extracts in the aqueous medium was evaluated in terms of the decrease in ABTS radical following a literature procedure with some changes [22]. Briefly, 2.0 mL of the aqueous solution 1.23 × 10^−4^ mol L^−1^ of the ABTS radical was added to 500 μL of aqueous solutions of each extract, and the mixture was kept in the dark for 6 min. The remaining ABTS concentration was spectrophotometrically evaluated at 734 nm, expressing the scavenging capacity in terms of IC_50_. Ascorbic acid was used as a positive control.

### 2.6. Effect of LWE3 on H_2_O_2_-Induced Reactive Oxygen Species Production in SH-SY5Y Cells

#### 2.6.1. Cell Cultures

Human SH-SY5Y neuroblastoma cells (ECACC Cat# 94030304, passages 7–20) were cultured in Dulbecco’s modified Eagle’s medium (DMEM), supplemented with 10% heat-inactivated fetal bovine serum (FBS), 100 U mL^−1^ penicillin, and 100 μg mL^−1^ streptomycin. Cell cultures were maintained in a humidified atmosphere at 37 °C and 5% CO_2_.

#### 2.6.2. Reactive Oxygen Species Detection

Intracellular reactive oxygen species (ROS) formation was investigated by using 2′,7′-dichlorofluorescein diacetate (DCF-DA). SH-SY5Y cells were seeded (2 × 10^4^ cells/well) into a 96-well plate, grown for 24 h under standard conditions, treated with LWE3 (10 and 25 mg mL^−1^) for 5 h at 37 °C, washed with PBS, and loaded with 10 µM DCF-DA for 30 min at 37 °C. The cells, rinsed twice with PBS, were treated with 2 mM H_2_O_2_ in DMEM without phenol red and the intracellular fluorescence was read immediately for 60 min with the Thermo Labsystems Synergy HTX reader (BioTek, Winooski, VT, USA) (485 nm excitation, 528 nm emission). The Area Under Curve_(0–60min)_ of the curve describing changes in fluorescence monitored every 5 min was calculated.

### 2.7. Statistical and Chemometric Analysis

HPLC-DAD analyses and antioxidant tests of the samples were performed in triplicate. Data were expressed as means ± SD and analyzed using the Wilcoxon test. A value of *p* < 0.05 was considered statistically significant. Biological experiment data are shown as the mean ± S.E.M. and analyzed by using ANOVA followed by the Tukey post-test with GraphPad Prism version 5.04 (GraphPad Software Inc., San Diego, CA, USA). In all comparisons, *p* < 0.05 was considered significant.

Principal component analysis (PCA) is an exploratory technique to extract the information contained in complex data systems, such as the spectral fingerprint of samples subjected to instrumental analysis. Samples and spectral variables are projected into the multidimensional space of the new principal components (PCs). PCA was performed to assess the composition differences in terms of the phenolic and flavonoid compounds of the wine lees extracts that can be detected by infrared spectral fingerprinting. The original data consisting of the extracts’ analysis were organized into a matrix, in which 108 samples were described by their FTIR-ATR spectra. PCA analysis was elaborated by the Singular Value Decomposition (SVD) algorithm and the full cross-validation (FCV) procedure allowed for the determination of the appropriate number of PCs [23].

Partial least square regression (PLS) is a factor analysis method for the elaboration of the multivariate model with predictive ability, where descriptor variables X and vector y containing the response variables are decomposed in orthogonal factors. Consecutive factors are evaluated until the covariance between descriptors and responses is maximized. PLS modeling is achieved when the factors that explain most of the covariation between both data sets are found. The number of factors is chosen by calculating the figures of the merit root mean square error of validation (RMSEV) and correlation coefficient R^2^ [24]. 

The multivariate analysis of the experimental data was carried out using the software Unscrambler X version 10.5 (CAMO, Trondheim, Norway).

## 3. Results and Discussion

An eco-friendly procedure was proposed to isolate antioxidant compounds from four different autochthones cultivars (*Magliocco Canino*, *Magliocco Rosato*, *Gaglioppo*, and *Nocera Rosso*) wine lees (Table 1). 

Specifically, water (pH = 2.0), ethanol, and a water (pH = 2.0)/ethanol (50/50 *v*/*v*) mixture were employed, while the extractions were performed at low temperature (30 °C) and reduced time (15 min) to preserve the structural integrity of the bioactive compounds. For all the analyzed cultivars, the extraction procedure performed by employing acidic water returned the best results in terms of yield (range 27.7–35.8%), while the hydroalcoholic mixture performance displayed values statistically (*p* < 0.05) reduced (13.2–20.2%). Finally, pure ethanol represented the worst extraction solvent for all the analyzed cultivars (2.4–6.3%). The recorded analytical results clearly highlight that winery by-products are rich in highly hydrophilic compounds, poorly interacting with the organic solvents. In all cases, the cultivar *Nocera Rosso* guarantees the highest yields. Focusing our attention on the phenolic molecules, the wine lees represent a vegetable matrix rich in high-added-value compounds, such as flavonoids, tannins, and phenolic acids [25]. The literature has established that the chemical integrity and biological characteristics of the active molecules can be successfully preserved by employing eco-friendly procedures, considering low temperatures, reduced extraction times, and green solvents [26]. Specifically, bioactive molecules were effectively extracted from the vegetal raw material by employing an ultrasound-assisted methodology, able to decrease both extraction time and temperature, avoiding the loss of the biological properties of the active compounds [27]. The chemical compositions and yields of the extracts are strictly related to the hydrophilicity of the solvent and the solid–solvent ratio. Usually, an increase in the solvent volume ensures a better swelling of the vegetal raw material, making easy the transfer of the polyphenols and enhancing the yield of the extraction procedure [28]. Additionally, the employment of a binary solvent system based on water and eco-friendly organic solvents was suggested by the literature and can guarantee high performance for the extraction of polyphenols from the vegetal matrix [29]. Specifically, the effect of ethanol concentration on the extraction yield was reported in the literature and the highest extraction yields of phenolic molecules were obtained when hydroalcoholic mixtures were used [30] due to the presence of a more polar medium increasing the polyphenol extraction efficiency [31]. In this regard, Kalia et al. found a 50% (*v*/*v*) ethanol solution in water to be the most effective for the extraction of phenolics from *Potentilla atrosanguinea* [32].

A detailed characterization of the extracts was performed by HPLC-DAD and FTIR spectroscopy.

### 3.1. HPLC-DAD Characterization of Polyphenols in Wine Lees Extracts

**Figure 1:**
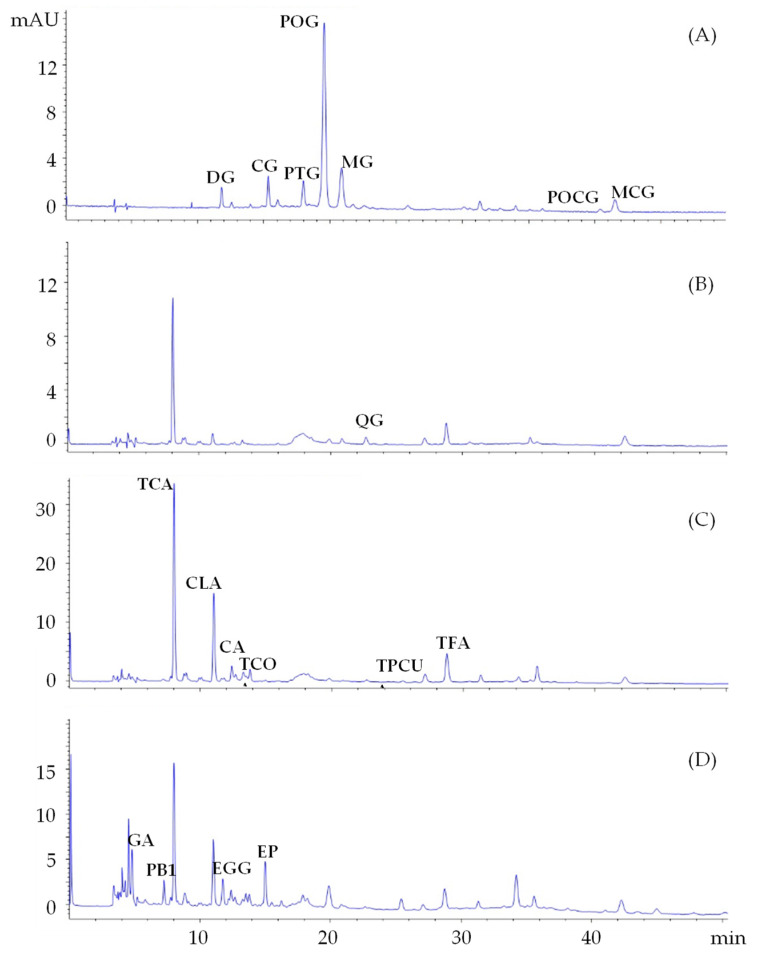
HPLC chromatograms registered at (**A**) 520 nm (DG = delphinidin-3*O*-glucoside; CG = cyanidin-3*O*-glucoside; PTG = petunidin-3*O*-glucoside; POG = peonidin-3*O*-glucoside; MG = malvidin-3*O*-glucoside; POCG = peonidin-3*O*-t-coumaroylglucoside; MCG = malvidin-3*O*-t-coumaroylglucoside), (**B**) 360 nm (QG = quercetin-3*O*-glucoside), (**C**) 320 nm (TCA = *trans*-caftaric acid; CLA = chlorogenic acid; CA = caffeic acid; TCOA = *trans*-coutaric acid; TPCUA = *trans*-p-coumaric acid; TFA = *trans*-ferulic acid), and (**D**) 280 nm (GA = gallic acid; PB1 = procyanidin B1; EG = (−)-epigallocatechin; PB2 = procyanidin B2; (−)-EGG = epigallocatechin gallate; EP = (−)-epicatechin).

Free phenolics are among the main constituents of wine lees [14]; therefore, HPLC-DAD analyses were performed to identify and quantify the main polyphenols in the different lees extracts (Figure 1), taking into consideration four wavelengths (280, 320, 360, and 520 nm) at which the absorption of flavan-3-ols and benzoic acids, hydroxycinnamic acids, flavonols, and anthocyanins, respectively, is maximum [33]. 

Starting from anthocyanins, seven compounds were unequivocally identified—delphinidin-3*O*-glucoside, cyanidin-3*O*-glucoside, petunidin-3*O*-glucoside, peonidin-3*O*-glucoside, and malvidin-3*O*-glucoside—because of the matching with commercial standards, while peonidin-3*O*-*trans*-coumaroylglucoside (POCG) and malvidin-3*O*-*trans*-coumaroylglucoside (MCG) were identified due to their characteristic chromatographic and spectrophotometric behavior many times reported in the literature [14,15]. Conversely, insufficient information was available to correctly identify the other anthocyanin conjugates in the extracts (Figure 1A).

Regarding their content, as expected [14], anthocyanins were more concentrated in lees from red wines (*Nocera Rosso* cv) and particularly in the water extracts due to the high polarity of these compounds; indeed, either the mono-glucoside anthocyanins or the two coumaroyl conjugates (POCG and MCG) were significantly more concentrated in LW1 than LWE1 and LE1 (Table 2).

Chlorogenic acid (CLA), caffeic acid (CA), *trans*-p-coumaric acid (TPCUA), and *trans*-ferulic acid (TFA) were revealed with the help of reference standards (Figure 1C). Generally, they were more concentrated in the hydroalcoholic extracts (LWE); in particular, the highest amounts of CLA (830 mg g^−1^), CA (180 mg g^−1^), TPCUA (46 mg g^−1^), and TFA (150 mg g^−1^) were quantified in *Magliocco Canino* (LWE3) and *Nocera Rosso* (LWE1), respectively (Table 2). It is worth noting that lower values of CLA, CA, and TPCUA were reported by Landeka et al. in methanolic (pH = 2) extracts of wine lees [34]. However, according to a previous report [14], the main hydroxycinnamic acid present in the extracts was *trans*-caftaric acid (TCA), reaching values of 2100 mg g^−1^ in LWE3 samples; this compound was tentatively identified together with *trans*-coutaric acid (TCOA) based on their typical UV spectra and chromatographic elution [14]. 

Gallic acid (GA) and five flavan-3-ols, namely procyanidin B_1_ (PB_1_), (−)-epigallocatechin (EG), procyanidin B_2_ (PB_2_), (−)-epigallocatechin gallate (EGG), and (−)-epicatechin (EP), showing variegated presence in the extracts, were also recognized (Figure 1D). In agreement with their polarity, the greater amounts of less hydrophilic PB1 (up to 700 mg g^−1^), PB2 (110 mg g^−1^), and EP (160 mg g^−1^) were found in the LWE extracts of *Nocera Rosso* and *Magliocco Canino*, whereas the more hydrophilic GA and EG were more concentrated in water extracts, specifically in LW1 (650 mg g^−1^) and LW2 (220 mg g^−1^), respectively. It is worth pointing out that the behavior of EGG was an exception because its values were prevalent in all LE extracts ranging from 820 to 2900 mg g^−1^ (Table 2). Finally, just quercetin-3*O*-glucoside (QG) was identified as a flavonol compound (Figure 1B) at higher levels in LWE1 and LWE3.

### 3.2. Characterization of Wine Lees Extracts Via FTIR and PCA

**Figure 2:**
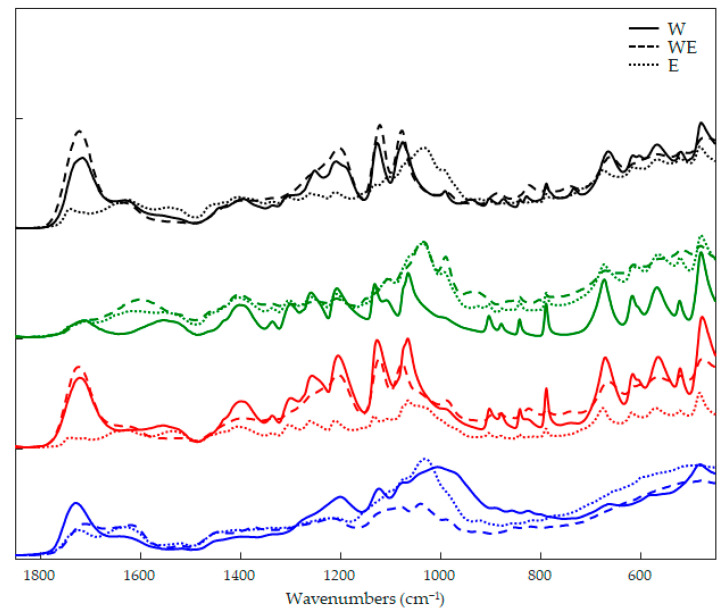
FTIR spectra for the different wine lees extractions: *Nocera Rosso* L1 (blue line), *Magliocco Rosato* L2 (red line), *Magliocco Canino* L3 (green line), and *Gaglioppo* L4 (black line), extracted by water (W, solid line), ethanol (E, dotted line), and hydroalcoholic (WE, dashed line) solutions.

The FTIR fingerprints of the wine lees are shown in Figure 2, where the variation in sample composition due to different cultivars and extraction strategies can be seen. The wavenumber window 1850–450 cm^−1^ presented the characteristic bands of phenolic compounds for all samples. The bands between 1470 and 950 cm^−1^ were due to the methoxy group of some phenolic acids. In the range of 1755–1630 cm^−1^, phenolic acids can be distinguished from flavonoids by the typical bands of the carboxyl groups, while flavonoid moieties are mainly distinguished by bands associated with benzo-γ-pyrone and benzopyrylium signals in the regions 1650–1400 and 1200–450 cm^−1^ [16].

**Figure 3:**
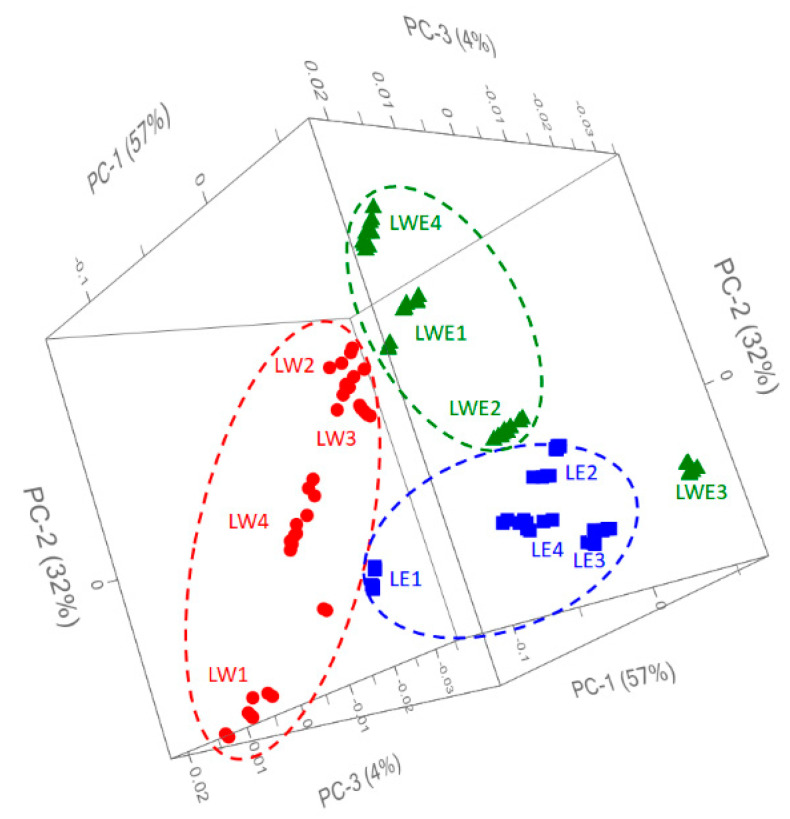
Three-dimensional score plot PC1-3 obtained by the PCA modeling of FTIR data from *Nocera Rosso* L1, *Magliocco Rosato* L2, *Magliocco Canino* L3, and *Gaglioppo* L4 extracts in the range 1850–450 cm^−1^ with a total explained variance of 93%.

Considering the intense overlapping of the signals from different compound families, a PCA was performed to investigate more specific differences in spectral fingerprints. Figure 3 describes the 3D plot scores by considering the principal components **1**, **2**, and **3** with a total explained variance of 93%. The clustering of the samples was immediately evident, and it was possible to distinguish the samples according to the type of solvent used to extract the phenolic compounds. The only exception was the sample LWE3, which stood out from the others by forming a distinct group. Analyzing the x-loading values, calculated for the processed wave variables with respect to the first three PCs, some characteristic peaks for each extraction technique or cultivar were highlighted.

PCA processing was able to handle and highlight a relative abundance of useful information in four spectral regions: 1736, 1720, 1390, and 1075 cm^−1^. The first band at 1736 cm^−1^ was apparently not responsible for the clustering of samples in the 3D-scores space; however, these characteristic bands could be attributed to the anthocyanins such as POCG and MCG, which have been detected for samples belonging to *Nocera Rosso* cultivar (L1) (Table 2 and Figure 2). Signals at 1720, 1120, and 1075 cm^−1^ were able to give enough information to allow the grouping of the samples and especially the clusters consisting of wine lees extracted by ethanol (LE) and hydroalcoholic (LWE) solvents. Chlorogenic acid and esters of caffeic acid showed bands around 1720 cm^−1^, which could be associated with the vibration of unsaturated aliphatic ester or the carbonyl stretching of protonated carboxylic acid; epigallocatechin gallate (EGG) is characterized by bands in the range 1120–1050 cm^−1^ and showed a band around 800 cm^−1^ [16]. Chromatographic analysis showed a relative abundance in the content of metabolites TCA, CA, CLA, and EGG for samples extracted in ethanol (LE) or hydroalcoholic (LWE) solvents, which was confirmed by evaluating the infrared fingerprint of the sample. The higher levels of these metabolites in the LWE3 sample were also evident in the infrared signals and were able to push the samples out of the LWE cluster by forming a distinct cluster.

### 3.3. Antioxidant Characterization of the Wine Lees Extracts

The total polyphenol content of wine lees extracts was accomplished according to the Folin–Ciocalteu methodology and the obtained results are reported in Table 3. The literature data reported that the phenolic profile in lees is strictly related to the type of crushed grapes and other factors that are present during vinification [8].

The recorded data displayed as the cultivar is an important parameter to be considered and deeply influences the performance of the extraction mixture. Ethanolic extracts returned the highest TPC values for *Magliocco Rosato* and *Magliocco Canino* cultivars, while the acidic water extraction of *Nocera Rosso* significantly guaranteed the best performance. Finally, the hydroalcoholic extract of *Magliocco Canino* displayed the highest TPC value (49.56 mg GA per g of extract) according to the data recovered by HPLC-DAD analysis. Recorded TPC value is in the same order of magnitude reported in the literature and related to wine lees deriving from different grape varieties and performing the extraction by several analytical methodologies. In particular, solvents with different polarities were also evaluated in the analyses of *Tempranillo* wines lees, returning TPC values in the range of 26 (acetone)-254 (ethanol/water 25:75 *v*/*v*) mg GA per gram of dry matter [35], according to another study performed a conventional extraction procedure and reporting a phenolic concentration equal to 547 mg GA per gram of dry matter [7]. Additionally, the TPC values of different extracts deriving from various red wines (*Cabernet Sauvignon*, *Cabernet Franc*, *Merlot*) by ultrasound were in the range of 44.02–58.71 mg GA per gram of dry extract [30], while other studies displayed decreased values of 23.2–30.9 mg GA per gram of dry extract [34,36]. On the other hand, these partial discrepancies can be largely due to the wine lees types, strictly related to the vinification process and grape variety as well as the extraction procedure.

LWE3 also showed the greatest concentration of flavonoid (20.15 mg GA per g of extract) and phenolic acid (22.67 mg CT per g of extract) compounds. Concerning the phenolic acids, *p*-coumaric and caffeic acids as well as their derivatives such as *trans*-coutaric acid and *trans*-caftaric acid, respectively, have been found in wine lees samples, according to the literature data reporting *trans*-caftaric acid as the predominant compounds in both *Vitis vinifera* and *non-vinifera* types [36].

Polyphenol moieties display numerous beneficial effects such as a remarkable scavenger ability against various radical species [37]. As antioxidant bio-compounds explain their action through different mechanisms, usually, various tests should be explored to fully estimate the antioxidant capacity of a compound mixture [38]. For this reason, the antioxidant profile of the extracts was evaluated in terms of scavenger activity both in aqueous and organic environments against DPPH and ABTS radicals, respectively. The capacity of the extracts to inhibit the lipophilic DPPH radical, expressed in terms of IC_50_ (mg mL^−1^), is reported in Table 3. The recorded results are in the range of 0.395–1.360 mg mL^−1^ and LWE3 appears as the more active extract. This trend was also established in the hydrophilic environment against the ABTS radical (IC_50_ for LWE3 equal to 0.451 mg mL^−1^), highlighting a greater scavenger effect of the extract in the organic environment with respect to the aqueous one. In general, scavenger activity measurements confirmed the TPC, PAC, and FC values. Specifically, a direct correlation between the antioxidant capacity of the extracts was carried out, verifying the existence of a direct dependence between TPC value and the scavenger activity, considering the sum of the contributions of the various antioxidant compounds in the sample and their possible synergistic effects. 

### 3.4. Effect of LWE3 on H_2_O_2_-Induced ROS Production in SH-SY5Y Cells

**Figure 4:**
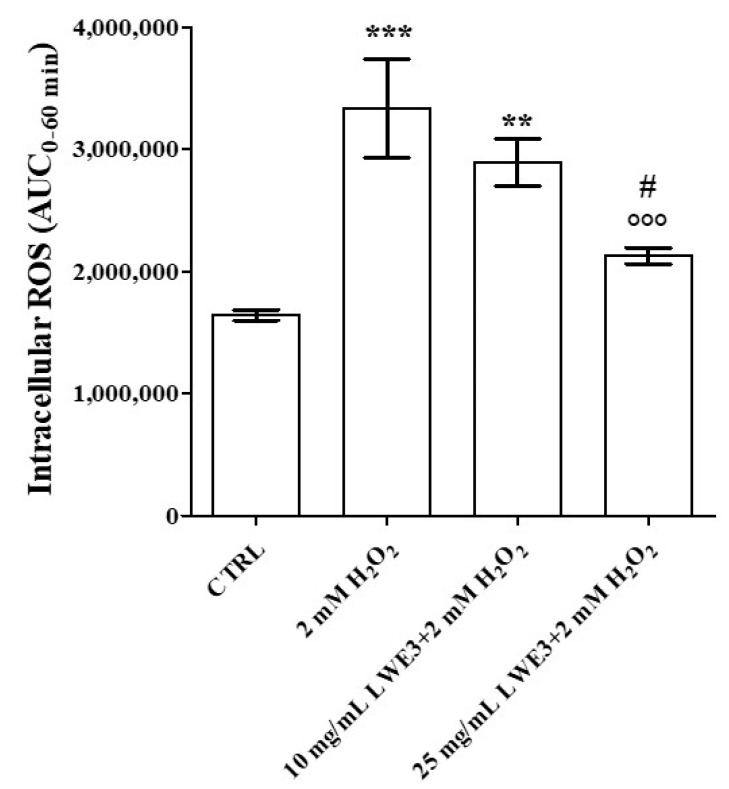
Total amount of intracellular reactive oxygen species in the control condition (CTRL) in presence of 2 mM H_2_O_2_, of 10 mg mL^−1^ LWE3 plus 2 mM H_2_O_2_ and of 25 mg mL^−1^ LWE3 plus 2 mM H_2_O_2_ during 60 min, expressed as Area Under Curve_(0–60 min)_. Data are reported as the mean ± S.E.M. of at least 3 independent experiments. LWE3 = lees of *Magliocco Canino* in water (pH = 2)/ethanol. *** *p* < 0.001 vs. CTRL, ** *p* < 0.01 vs. CTRL, °°° *p* < 0.001 vs. H_2_O_2_, # *p* < 0.05 vs. 10 mg mL^−1^ LWE3 + 2 mM H_2_O_2_, ANOVA followed by Tukey post-test.

In SH-SY5Y cells, treatment with H_2_O_2_ markedly increased the intracellular level of ROS (Figure 4). However, pre-treatment with LWE3 significantly prevented ROS production in a concentration-dependent manner, suggesting that the extract protected neuroblastoma cells against oxidative stress damage.

### 3.5. Relationship between Antioxidant Properties and Metabolite Composition

In the present study, the PLS algorithm was applied to study the relationship between antioxidant properties and the metabolite composition of the extracts. The root mean square error of validation (RMSEV) and the correlation coefficient R^2^ were used to evaluate the fitting of the multivariate models with the experimental results. Usually, an R^2^ value higher than 0.5 is indicative of an acceptable model and a value over 0.8 proves an excellent correlation [39]. 

The chemical composition detected by HPLC analysis was used as a descriptor variable (matrix X) for each sample, and two different response vectors, ***y_DPPH_*** and ***y_ABTS_***, consisting of the respective IC_50_ values evaluated for each sample, were selected. All variables used in the building of the model were re-scaled and standardized prior to elaboration; they were normalized by dividing by the standard deviation value in the original scale and in logarithmic form.

**Figure 5:**
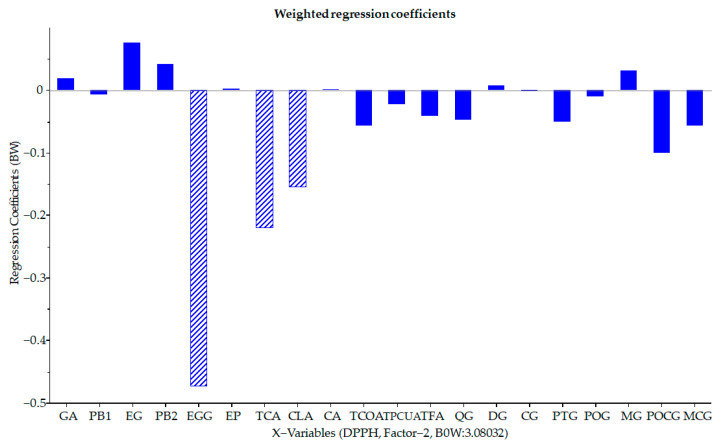
The weighted regression coefficients (Bw) were calculated for all the x-variables used in PLS calibration for the prediction of DPPH IC_50_ (***y_DPPH_***).

First, multivariate calibration built two PLS models with very low predictive performance with R^2^ below 0.6 for both ***y_DPPH_*** and ***y_ABTS_***. These models were used as an initial screening of the correlation between the compound concentrations and the antioxidant power of the extracts. After evaluating the regression coefficients of each variable, it was possible to determine which of them provided the most information in the calibration of the models. Figure 5 shows the weighted regression coefficients (Bw) plot. Regression coefficients describe the relationship between all the descriptors (x-variables) and a given response (y-variable). Bw coefficients provide information about the importance of the x-variables: a high Bw value means an important role in the building of the models, while x-variables with small coefficients may be negligible; a positive coefficient shows a direct proportionality with the response, and a negative coefficient shows an indirect proportionality. The most important variables were TCA, CLA, and EGG for the prediction of ***y_DPPH_*** and TCA and CLA for the prediction of ***y_ABTS_***. 

A new calibration procedure was thus optimized by considering just the variables TCA, CLA, and EGG. A significant improvement in the statistical parameters was observed when the concentration levels of phenolic acids and flavonoids were considered on the logarithmic scale. The PLS model built to predict the DPPH IC_50_ gave good statistical parameters with a correlation coefficient R^2^ value of 0.932 and a prediction error RMSEV of 0.023 by using three factors, while ABTS IC_50_ was correlated with R^2^ of 0.824 an RMSEV of 0.064 by using two factors.

## 4. Conclusions

In this study, wine lees from different cultivars (*Magliocco Canino*, *Magliocco Rosato*, *Gaglioppo,* and *Nocera Rosso*) were extracted with an ultrasound-assisted method using an *eco*-friendly procedure employing low temperature and reduced time and ethanol and water as solvents to obtain polyphenolic fractions deeply analyzed by HPLC-DAD and FTIR techniques. Specifically, anthocyanins were more concentrated in lees from red winemaking (*Nocera Rosso cv*) and particularly in the water extracts due to the high polarity of these compounds, while the main hydroxycinnamic acid present in the extracts was *trans*-caftaric acid, reaching values of 2100 mg g^−1^ in LWE3 samples. Additionally, colorimetric tests allowed for the evaluation of total polyphenol, phenolic acid, and flavonoid contents, while radical scavenging assays were performed to quantify the antioxidant performances of the extracts, both in organic and aqueous media, against DPPH and ABTS radicals, respectively. The combined use of multivariate tools and FTIR spectral analysis proved very effective in characterizing samples from different cultivars and extraction procedures, grouping samples with respect to their spectral fingerprint, and highlighting the correspondence between spectral bands and metabolite abundance in extracts. The collected data confirmed the spectroscopic recovery, highlighting LWE3 as representing the higher-performing extract. Cellular (SH-SY5Y cells) antioxidant assays were performed on the best extract (LWE3) to confirm its biological performance against radical species.

The chemometric processing of the qualitative–quantitative experimental data allowed the correlations between chemical composition and antioxidant features of the different wine lees extracts to be found. PLS optimization identified the metabolites responsible for the detected antioxidant effects, and two PLS models were built to predict the IC_50_ values of DPPH and ABTS radicals with good predictive ability by using the concentration values of the compounds TCA, CLA, and EGG.

Further studies will employ the best extract to prepare innovative high-value functional foods. Alternative antioxidant extracts will be involved in a radical conjugation process to synthesize more stable macromolecular systems able to be used as an additive in the food industry. 

## Figures and Tables

**Table 1:** Ultrasound-assisted extractions of wine lees from different cultivars.

Sample		Extraction Conditions				Yield	
*Code*	*Mass Dry Extract* (g)	*Solvent*	*Volume* (mL)	*T* (°C)	*t* (min)	*Mass* (g)	*%*
**LE1**	1.0	Ethanol	200	30	15	0.063 ± 0.005 ^c^	6.3 ± 0.5 ^ca^
**LEW1**	1.0	Water (pH = 2.0)/Ethanol 50:50 *v*/*v*	200	30	15	0.202 ± 0.014 ^f^	20.2 ± 1.5 ^f^
**LW1**	1.0	Water (pH = 2.0)	200	30	15	0.358 ± 0.007 ^i^	35.8 ± 0.6 ^i^
**LE2**	1.0	Ethanol	200	30	15	0.025 ± 0.001 ^a^	2.5 ± 0.1 ^a^
**LEW2**	1.0	Water (pH = 2.0)/Ethanol 50:50 *v*/*v*	200	30	15	0.132 ± 0.006 ^d^	13.2 ± 0.5 ^d^
**LW2**	1.0	Water (pH = 2.0)	200	30	15	0.277 ± 0.008 ^g^	27.7 ± 0.7 ^g^
**LE3**	1.0	Ethanol	200	30	15	0.040 ± 0.003 ^b^	4.0 ± 0.3 ^b^
**LEW3**	1.0	Water (pH = 2.0)/Ethanol 50:50 *v*/*v*	200	30	15	0.137 ± 0.014 ^d^	13.7 ± 1.5 ^d^
**LW3**	1.0	Water (pH = 2.0)	200	30	15	0.313 ± 0.007 ^h^	31.3 ± 0.6 ^h^
**LE4**	1.0	Ethanol	200	30	15	0.024 ± 0.001 ^a^	2.4 ± 0.1 ^a^
**LEW4**	1.0	Water (pH = 2.0)/Ethanol 50:50 *v*/*v*	200	30	15	0.169 ± 0.006 ^e^	16.9 ± 0.5 ^e^
**LW4**	1.0	Water (pH = 2.0)	200	30	15	0.278 ± 0.008 ^g^	27.8 ± 0.7 ^g^

LE1 = lees of *Nocera Rosso* in ethanol; LWE1 = lees of *Nocera Rosso* in water (pH = 2)/ethanol; LW1 = l lees of *Nocera Rosso* in water (pH = 2); LE2 = lees of *Magliocco Rosato* in ethanol; LWE2 = lees of *Magliocco Rosato* in water (pH = 2)/ethanol; LW2 = lees of *Magliocco Rosato* in water (pH = 2); LE3 = lees of *Magliocco Canino* in ethanol; LWE3 = lees of *Magliocco Canino* in water (pH = 2)/ethanol; LW3 = lees of *Magliocco Canino* in water (pH = 2); LE4 = lees of *Gaglioppo* in ethanol; LWE4 = lees of *Gaglioppo* in water (pH = 2)/ethanol; LW4 = lees of *Gaglioppo* in water (pH = 2). Different letters in the same column express significant differences (*p* < 0.05).

**Table 2:** Content (expressed in mg g^−1^) of the identified polyphenols into the wine lees extracts.

Compound	RT (min)	LE1	LWE1	LW1	LE2	LWE2	LW2	LE3	LWE3	LW3	LE4	LWE4	LW4
*λ = 280* nm													
Gallic acid	4.733	n.d.	390 ± 60 ^b^	650 ± 90 ^a^	n.d.	0.39 ± 0.04 ^d^	19 ± 3 ^c^	n.d.	n.d.	tr	n.d.	34 ± 6 ^c^	15 ± 3 ^c^
Procyanidin B_1_	7.124	tr	180 ± 40 ^b^	44 ± 8 ^c^	n.d.	n.d.	n.d.	n.d.	700 ± 140 ^a^	n.d.	tr	tr	n.d.
Unknown	7.745	n.d.	n.d.	n.d.	n.d.	n.d.	460 ± 90 ^ab^	340 ± 60 ^b^	620 ± 120 ^a^	tr	140 ± 20 ^c^	180 ± 40 ^c^	400 ± 60 ^ab^
(−)-epigallocatechin	8.643	n.d.	n.d.	n.d.	n.d.	n.d.	220 ± 50 ^a^	n.d.	n.d.	n.d.	n.d.	160 ± 30 ^b^	110 ± 20 ^b^
Procyanidin B_2_	10.699	n.d.	n.d.	n.d.	n.d.	n.d.	21 ± 3 ^b^	n.d.	110 ± 20 ^a^	n.d.	tr	tr	12.8 ± 1.9 ^b^
Epigallocatechin gallate	11.736	820 ± 160 ^c^	250 ± 50 ^d^	n.d.	1800 ± 400 ^b^	190 ± 40 ^d^	n.d.	2900 ± 700 ^a^	1200 ± 300 ^b^	n.d.	880 ± 190 ^c^	260 ± 50 ^d^	n.d.
(−)-Epicatechin	13.544	n.d.	120 ± 20 ^a^	34 ± 5 ^b^	n.d.	n.d.	n.d.	n.d.	n.d.	n.d.	tr	tr	5.5 ± 0.8 ^c^
Unknown	14.795	210 ± 40 ^b^	370 ± 80 ^ab^	110 ± 20 ^c^	n.d.	n.d.	n.d.	n.d.	410 ± 90 ^a^	n.d.	n.d.	n.d.	n.d.
Unknown	16.224	n.d.	40 ± 7	n.d.	n.d.	n.d.	n.d.	n.d.	n.d.	n.d.	n.d.	n.d.	n.d.
Unknown	25.406	n.d.	160 ± 30	n.d.	n.d.	n.d.	n.d.	n.d.	n.d.	n.d.	n.d.	n.d.	n.d.
*λ = 320* nm													
Unknown	6.363	n.d.	n.d.	n.d.	n.d.	n.d.	n.d.	n.d.	170 ± 40 ^a^	n.d.	20 ± 3 ^b^	5.8 ± 0.4 ^c^	1.37 ± 0.14 ^c^
Unknown	7.081	n.d.	n.d.	n.d.	56 ± 12 ^a^	42 ± 8 ^ab^	26 ± 5 ^b^	n.d.	51 ± 10 ^a^	n.d.	n.d.	7.2 ± 0.8 ^c^	12 ± 2 ^bc^
*trans*-caftaric acid	7.899	710 ± 140 ^c^	570 ± 120 ^cd^	330 ± 70 ^d^	1200 ± 300 ^b^	500 ± 100 ^cd^	220 ± 40 ^b^	170 ± 30 ^e^	2100 ± 400 ^a^	48 ± 8 ^b^	520 ± 90 ^cd^	230 ± 40 ^de^	250 ± 50 ^d^
Unknown	8.380	n.d.	n.d.	87 ± 19 ^a^	n.d.	7.8 ± 0.9 ^b^	1.9 ± 0.4 ^b^	n.d.	n.d.	n.d.	n.d.	tr	n.d.
Unknown	8.625	27 ± 6 ^b^	20 ± 4 ^b^	20 ± 3 ^b^	tr	13 ± 3 ^c^	n.d.	n.d.	66 ± 9 ^a^	tr	n.d.	tr	3.2 ± 0.7 ^d^
Unknown	8.774	29 ± 6 ^b^	31 ± 8 ^b^	41 ± 7 ^ab^	n.d.	21 ± 5 ^b^	5.2 ± 1.1 ^c^	n.d.	72 ± 14 ^a^	tr	n.d.	3.2 ± 0.7 ^c^	1.5 ± 0.3 ^c^
Chlorogenic acid	10.871	370 ± 70 ^b^	290 ± 60 ^b^	170 ± 30 ^bc^	230 ± 50 ^b^	102 ± 14 ^c^	60 ± 12 ^d^	66 ± 13 ^d^	830 ± 140 ^a^	27 ± 5 ^e^	130 ± 30 ^c^	55 ± 10 ^d^	51 ± 10 ^d^
Unknown	11.41	11 ± 3 ^b^	9 ± 2 ^b^	11 ± 3 ^b^	n.d.	n.d.	n.d.	n.d.	25 ± 6 ^a^	n.d.	n.d.	n.d.	n.d.
Unknown	12.403	16 ± 3 ^bc^	50 ± 9 ^b^	n.d.	tr	4.8 ± 0.9 ^c^	8.4 ± 1.5 ^c^	n.d.	160 ± 40 ^a^	n.d.	n.d.	2.6 ± 0.5 ^c^	3.4 ± 0.6 ^c^
Unknown	12.691	16 ± 4 ^a^	21 ± 5 ^a^	n.d.	n.d.	n.d.	n.d.	n.d.	n.d.	n.d.	12 ± 4 ^a^	n.d.	n.d.
Caffeic acid	13.097	n.d.	35 ± 8 ^b^	25 ± 5 ^b^	tr	12 ± 3 ^b^	5.6 ± 1.2 ^c^	n.d.	180 ± 50 ^a^	5.0 ± 1.1 ^c^	20 ± 4 ^b^	7.5 ± 1.8 ^c^	7.5 ± 1.4 ^c^
*trans*-coutaric acid	13.794	10 ± 2 ^b^	37 ± 10 ^a^	n.d.	n.d.	n.d.	n.d.	n.d.	16 ± 4 ^b^	n.d.	n.d.	n.d.	n.d.
*trans*-p-coumaric acid	27.072	n.d.	46 ± 9	n.d.	n.d.	n.d.	n.d.	n.d.	n.d.	n.d.	n.d.	n.d.	n.d.
*trans*-ferulic acid	28.728	n.d.	150 ± 30 ^a^	n.d.	n.d.	33 ± 9 ^b^	n.d.	n.d.	n.d.	n.d.	n.d.	n.d.	n.d.
Unknown	31.306	n.d.	31 ± 7	n.d.	n.d.	n.d.	n.d.	n.d.	n.d.	n.d.	n.d.	n.d.	n.d.
Unknown	34.182	n.d.	18 ± 3	n.d.	n.d.	n.d.	n.d.	n.d.	n.d.	n.d.	n.d.	n.d.	n.d.
Unknown	35.586	n.d.	69 ± 15	n.d.	n.d.	n.d.	n.d.	n.d.	n.d.	n.d.	n.d.	n.d.	n.d.
*λ = 360* nm													
Quercetin-3*O*-glucoside	22.627	tr	26 ± 6 ^b^	n.d.	n.d.	8.3 ± 1.8 ^bc^	n.d.	n.d.	90 ± 20 ^a^	n.d.	25 ± 5 ^b^	4.5 ± 1.0 ^c^	4.70 ± 0.8 ^c^
*λ = 520* nm													
Delphinidin-3*O*-glucoside	11.782	16 ± 4 ^b^	n.d.	86 ± 18 ^a^	n.d.	n.d.	n.d.	n.d.	n.d.	n.d.	n.d.	n.d.	n.d.
Cyanidin -3*O*-glucoside	15.303	30 ± 7 ^b^	12 ± 3 ^b^	135 ± 30 ^a^	n.d.	n.d.	n.d.	n.d.	n.d.	n.d.	n.d.	n.d.	n.d.
Petunidin -3*O*-glucoside	17.959	52 ± 11 ^ab^	20 ± 4 ^b^	87 ± 16 ^a^	n.d.	n.d.	n.d.	n.d.	n.d.	n.d.	n.d.	n.d.	n.d.
Peonidin-3*O*-glucoside	19.531	400 ± 90 ^b^	190 ± 40 ^b^	1400 ± 300 ^a^	n.d.	16 ± 3 ^c^	n.d.	n.d.	n.d.	n.d.	n.d.	n.d.	n.d.
Malvidin-3 *O*-glucoside	20.843	n.d.	9.0 ± 1.9 ^b^	330 ± 70 ^a^	n.d.	5.3 ± 1.3 ^b^	n.d.	n.d.	n.d.	n.d.	n.d.	n.d.	n.d.
Unknown	21.744	n.d.	n.d.	24 ± 7	n.d.	n.d.	n.d.	n.d.	n.d.	n.d.	n.d.	n.d.	n.d.
Unknown	22.549	n.d.	n.d.	33 ± 8	n.d.	n.d.	n.d.	n.d.	n.d.	n.d.	n.d.	n.d.	n.d.
Unknown	25.853	n.d.	n.d.	21 ± 4	n.d.	n.d.	n.d.	n.d.	n.d.	n.d.	n.d.	n.d.	n.d.
Unknown	31.293	n.d.	n.d.	52 ± 12	n.d.	n.d.	n.d.	n.d.	n.d.	n.d.	n.d.	n.d.	n.d.
Unknown	33.991	n.d.	n.d.	22 ± 5	n.d.	n.d.	n.d.	n.d.	n.d.	n.d.	n.d.	n.d.	n.d.
Peonidin-3*O*-*trans*-coumaroylglucoside	40.364	31 ± 7 ^a^	11 ± 3 ^b^	24 ± 5 ^a^	n.d.	n.d.	n.d.	n.d.	n.d.	n.d.	n.d.	n.d.	n.d.
Malvidin-3 *O*-*trans*-coumaroylglucoside	41.482	72 ± 13 ^b^	79 ± 15 ^b^	110 ± 20 ^a^	n.d.	n.d.	n.d.	n.d.	n.d.	n.d.	n.d.	n.d.	n.d.
**Total**		2820 ± 623	3045 ± 642	3802 ± 749 ^b^	3286 ± 741	929 ± 180	1026 ± 196	3476 ± 685 ^b^	6800 ± 1445 ^a^	75 ± 14	1747 ± 358	919 ± 167	838 ± 145

n.d. = not detected; tr = trace. RT = retention time. LE1 = lees of *Nocera Rosso* in ethanol; LWE1 = lees of *Nocera Rosso* in water/ethanol; LW1 = lees of *Nocera Rosso* in water; LE2 = lees of *Magliocco Rosato* in ethanol; LWE2 = lees of *Magliocco Rosato* in water/ethanol; LW2 = lees of *Magliocco Rosato* in water; LE3 = lees of *Magliocco Canino* in ethanol; LWE3 = lees of *Magliocco Canino* in water/ethanol; LW3 = lees of *Magliocco Canino* in water; LE4 = lees of *Gaglioppo* in ethanol; LWE4 = lees of *Gaglioppo* in water/ethanol; LW4 = lees of *Gaglioppo* in water. Different letters in the same row express significant differences (*p* < 0.05) (Duncan test).

**Table 3:** Total phenolic content, phenolic acid content, flavonoid content, and antioxidant activity of the extracts from wine lees.

Sample	TPC (mg *GA* g^−1^ *extract*)	PAC (mg *GA* g^−1^ *extract*)	FC (mg *CT* g^−1^ *extract*)	IC_50_ (mg mL^−1^)	
				*DPPH*	*ABTS*
**LE1**	18.94 ± 0.37 ^e^	5.89 ± 0.15 ^f^	9.54 ± 0.17 ^f^	0.470 ± 0.017 ^c^	0.770 ± 0.027 ^d^
**LWE1**	20.01 ± 0.46 ^f^	6.58 ± 0.16 ^g^	10.89 ± 0.26 ^g^	0.490 ± 0.022 ^c^	0.690 ± 0.025 ^c^
**LW1**	27.23 ± 0.49 ^h^	10.25 ± 0.49 ^l^	18.15 ± 0.38 ^l^	0.608 ± 0.019 ^e^	0.500 ± 0.019 ^b^
**LE2**	24.25 ± 0.47 ^g^	9.14 ± 0.17 ^h^	13.23 ± 0.17 ^h^	0.457 ± 0.022 ^c^	0.790 ± 0.027 ^d^
**LWE2**	11.02 ± 0.36 ^c^	3.12 ± 0.08 ^c^	7.59 ± 0.16 ^d^	0.643 ± 0.025 ^e^	0.710 ± 0.030 ^c^
**LW2**	10.89 ± 0.29 ^c^	3.26 ± 0.10 ^c^	8.25 ± 0.19 ^e^	0.803 ± 0.038 ^f^	0.680 ± 0.032 ^c^
**LE3**	23.72 ± 0.27 ^g^	8.89 ± 0.17 ^h^	16.24 ± 0.26 ^i^	0.419 ± 0.013 ^a^	0.700 ± 0.027 ^c^
**LWE3**	49.56 ± 0.56 ^i^	22.67 ± 0.56 ^i^	20.15 ± 0.32 ^m^	0.395 ± 0.016 ^a^	0.451 ± 0.015 ^a^
**LW3**	4.61 ± 0.09 ^a^	1.58 ± 0.07 ^a^	1.54 ± 0.07 ^a^	1.360 ± 0.067 ^h^	1.480 ± 0.049 ^g^
**LE4**	12.24 ± 0.22 ^d^	5.54 ± 0.12 ^e^	6.25 ± 0.22 ^c^	0.560 ± 0.028 ^d^	0.850 ± 0.032 ^ef^
**LWE4**	11.35 ± 0.26 ^c^	2.54 ± 0.08 ^b^	8.01 ± 0.16 ^e^	0.630 ± 0.026 ^e^	0.800 ± 0.026 ^de^
**LW4**	10.29 ± 0.23 ^b^	2.65 ± 0.07 ^b^	5.23 ± 0.13 ^b^	1.080 ± 0.050 ^g^	0.912 ± 0.035 ^f^
*Positive control*					
*Ascorbic acid*				0.017 ± 0.002	0.050 ± 0.002

TPC = total phenolic content; PAC = phenolic acid content; FC = flavonoid content; GA = gallic acid; CT = catechin; DPPH = (2.2-diphenyl-1-picrylhydrazyl); ABTS = (2.2′-azinobis (3-ethylbenzothiazoline-6-sulphonic acid)); LE1 = lees of *Nocera Rosso* in ethanol; LWE1 = lees of *Nocera Rosso* in water (pH = 2)/ethanol; LW1 = l lees of *Nocera Rosso* in water (pH = 2); LE2 = lees of *Magliocco Rosato* in ethanol; LWE2 = lees of *Magliocco Rosato* in water (pH = 2)/ethanol; LW2 = lees of *Magliocco Rosato* in water (pH = 2); LE3 = lees of *Magliocco Canino* in ethanol; LWE3 = lees of *Magliocco Canino* in water (pH = 2)/ethanol; LW3 = lees of *Magliocco Canino* in water (pH = 2); LE4 = lees of *Gaglioppo* in ethanol; LWE4 = lees of *Gaglioppo* in water (pH = 2)/ethanol; LW4 = lees of *Gaglioppo* in water (pH = 2). Each measurement was carried out in triplicate and data are expressed as means (±SD). Different letters express significant differences (*p* < 0.05).

## Data Availability

The data presented in this study are available on request from the corresponding author. The data are not publicly available as they contain information that could compromise the privacy of research participants.

## References

[1] Moscovici D., Gow J., Alonso Ugaglia A., Rezwanul R., Valenzuela L., Mihailescu R. (2022). Consumer Preferences for Organic Wine-Global Analysis of People and Place. J. Clean Prod..

[2] Sellers R., Alampi-Sottini V. (2016). The Influence of Size on Winery Performance: Evidence from Italy. Wine Econ. Policy.

[3] Lavelli V., Sri Harsha P.S.C., Ferranti P., Scarafoni A., Iametti S. (2016). Grape Skin Phenolics as Inhibitors of Mammalian α-Glucosidase and α-Amylase-Effect of Food Matrix and Processing on Efficacy. Food Funct..

[4] González-Paramás A.M., Esteban-Ruano S., Santos-Buelga C., de Pascual-Teresa S., Rivas-Gonzalo J.C. (2004). Flavanol Content and Antioxidant Activity in Winery Byproducts. J. Agric. Food Chem..

[5] Restuccia D., Giorgi G., Gianfranco Spizzirri U., Sciubba F., Capuani G., Rago V., Carullo G., Aiello F. (2019). Autochthonous White Grape Pomaces as Bioactive Source for Functional Jams. Int. J. Food Sci. Technol..

[6] Ruggieri L., Cadena E., Martínez-Blanco J., Gasol C.M., Rieradevall J., Gabarrell X., Gea T., Sort X., Sánchez A. (2009). Recovery of Organic Wastes in the Spanish Wine Industry. Technical, Economic and Environmental Analyses of the Composting Process. J. Clean Prod..

[7] Pérez-Serradilla J.A., Luque de Castro M.D. (2011). Microwave-Assisted Extraction of Phenolic Compounds from Wine Lees and Spray-Drying of the Extract. Food Chem..

[8] Jara-Palacios M.J. (2019). Wine Lees as a Source of Antioxidant Compounds. Antioxidants.

[9] Barcia M.T., Pertuzatti P.B., Gómez-Alonso S., Godoy H.T., Hermosín-Gutiérrez I. (2014). Phenolic Composition of Grape and Winemaking By-Products of Brazilian Hybrid Cultivars BRS Violeta and BRS Lorena. Food Chem..

[10] Irimia L.M., Patriche C.V., Murariu O.C. (2018). The Impact of Climate Change on Viticultural Potential and Wine Grape Varieties of a Temperate Wine Growing Region. Appl. Ecol. Environ. Res..

[11] Versari A., Castellari M., Spinabelli U., Galassi S. (2001). Recovery of Tartaric Acid from Industrial Enological Wastes. J. Chem. Technol. Biotechnol..

[12] Alarcón M., López-Viñas M., Pérez-Coello M.S., Díaz-Maroto M.C., Alañón M.E., Soriano A. (2020). Effect of Wine Lees as Alternative Antioxidants on Physicochemical and Sensorial Composition of Deer Burgers Stored during Chilled Storage. Antioxidants.

[13] Carullo G., Durante M., Sciubba F., Restuccia D., Spizzirri U.G., Ahmed A., di Cocco M.E., Saponara S., Aiello F., Fusi F. (2019). Vasoactivity of Mantonico and Pecorello Grape Pomaces on Rat Aorta Rings: An Insight into Nutraceutical Development. J. Funct. Foods.

[14] Matos M.S., Romero-Díez R., Álvarez A., Bronze M.R., Rodríguez-Rojo S., Mato R.B., Cocero M.J., Matias A.A. (2019). Polyphenol-Rich Extracts Obtained from Winemakingwaste Streams as Natural Ingredients with Cosmeceutical Potential. Antioxidants.

[15] Alba V., Natrella G., Gambacorta G., Crupi P., Coletta A. (2022). Effect of over Crop and Reduced Yield by Cluster Thinning on Phenolic and Volatile Compounds of Grapes and Wines of ‘Sangiovese’ Trained to Tendone. J. Sci. Food Agric..

[16] Abbas O., Compère G., Larondelle Y., Pompeu D., Rogez H., Baeten V. (2017). Phenolic Compound Explorer: A Mid-Infrared Spectroscopy Database. Vib. Spectrosc..

[17] Ceramella J., la Torre C., De Luca M., Iacopetta D., Fazio A., Catalano A., Ragno G., Longo P., Sinicropi M.S., Rosano C. (2022). Exploring the Anticancer and Antioxidant Properties of *Vicia faba* L. Pods Extracts, a Promising Source of Nutraceuticals. PeerJ.

[18] Spizzirri U.G., Carullo G., de Cicco L., Crispini A., Scarpelli F., Restuccia D., Aiello F. (2019). Synthesis and Characterization of a (+)-Catechin and L-(+)-Ascorbic Acid Cocrystal as a New Functional Ingredient for Tea Drinks. Heliyon.

[19] Spizzirri U.G., Caputo P., Oliviero Rossi C., Crupi P., Muraglia M., Rago V., Malivindi R., Clodoveo M.L., Restuccia D., Aiello F. (2022). A Tara Gum/Olive Mill Wastewaters Phytochemicals Conjugate as a New Ingredient for the Formulation of an Antioxidant-Enriched Pudding. Foods.

[20] Spizzirri U.G., Carullo G., Aiello F., Paolino D., Restuccia D. (2021). Valorisation of Olive Oil Pomace Extracts for a Functional Pear Beverage Formulation. Int. J. Food Sci. Technol..

[21] Spizzirri U.G., Abduvakhidov A., Caputo P., Crupi P., Muraglia M., Rossi C.O., Clodoveo M.L., Aiello F., Restuccia D. (2022). Kefir Enriched with Carob (*Ceratonia siliqua* L.) Leaves Extract as a New Ingredient during a Gluten-Free Bread-Making Process. Fermentation.

[22] Carullo G., Spizzirri U.G., Montopoli M., Cocetta V., Armentano B., Tinazzi M., Sciubba F., Giorgi G., Enrica Di Cocco M., Bohn T. (2022). Milk Kefir Enriched with Inulin-Grafted Seed Extract from White Wine Pomace: Chemical Characterisation, Antioxidant Profile and in Vitro Gastrointestinal Digestion. Int. J. Food Sci. Technol..

[23] Spatari C., De Luca M., Ioele G., Ragno G. (2017). A Critical Evaluation of the Analytical Techniques in the Photodegradation Monitoring of Edible Oils. LWT.

[24] De Luca M., Ioele G., Spatari C., Caruso L., Galasso M.P., Ragno G. (2019). Evaluation of Human Breastmilk Adulteration by Combining Fourier Transform Infrared Spectroscopy and Partial Least Square Modeling. Food Sci. Nutr..

[25] Chang Y., Shi X., He F., Wu T., Jiang L., Normakhamatov N., Sharipov A., Wang T., Wen M., Aisa H.A. (2022). Valorization of Food Processing Waste to Produce Valuable Polyphenolics. J. Agric. Food Chem..

[26] Cañadas R., González-Miquel M., González E.J., Díaz I., Rodríguez M. (2020). Overview of Neoteric Solvents as Extractants in Food Industry: A Focus on Phenolic Compounds Separation from Liquid Streams. Food Res..

[27] Montenegro-Landívar M.F., Tapia-Quirós P., Vecino X., Reig M., Valderrama C., Granados M., Cortina J.L., Saurina J. (2021). Fruit and Vegetable Processing Wastes as Natural Sources of Antioxidant-Rich Extracts: Evaluation of Advanced Extraction Technologies by Surface Response Methodology. J. Environ. Chem. Eng..

[28] Clodoveo M.L., Crupi P., Muraglia M., Corbo F. (2022). Ultrasound Assisted Extraction of Polyphenols from Ripe Carob Pods (*Ceratonia siliqua* L.): Combined Designs for Screening and Optimizing the Processing Parameters. Foods.

[29] Rached I., Barros L., Fernandes I.P., Santos-Buelga C., Rodrigues A.E., Ferchichi A., Barreiro M.F., Ferreira I.C.F.R. (2016). *Ceratonia siliqua* L. Hydroethanolic Extract Obtained by Ultrasonication: Antioxidant Activity, Phenolic Compounds Profile and Effects in Yogurts Functionalized with Their Free and Microencapsulated Forms. Food Funct..

[30] Tao Y., Wu D., Zhang Q.A., Sun D.W. (2014). Ultrasound-Assisted Extraction of Phenolics from Wine Lees: Modeling, Optimization and Stability of Extracts during Storage. Ultrason. Sonochem..

[31] de Bruno A., Romeo R., Fedele F.L., Sicari A., Piscopo A., Poiana M. (2018). Antioxidant Activity Shown by Olive Pomace Extracts. J. Environ. Sci. Health B.

[32] Kalia K., Sharma K., Singh H.P., Singh B. (2008). Effects of Extraction Methods on Phenolic Contents and Antioxidant Activity in Aerial Parts of Potentilla Atrosanguinea Lodd. and Quantification of Its Phenolic Constituents by RP-HPLC. J. Agric. Food Chem..

[33] Crupi P., Antonacci D., Savino M., Genghi R., Perniola R., Coletta A. (2016). Girdling and Gibberellic Acid Effects on Yield and Quality of a Seedless Red Table Grape for Saving Irrigation Water Supply. Eur. J. Agron..

[34] Jurcevic I.L., Dora M., Guberovic I., Petras M., Brncic S.R., Dikic D. (2017). Polyphenols from Wine Lees as a Novel Functional Bioactive Compound in the Protection against Oxidative Stress and Hyperlipidaemia. Food Technol. Biotechnol..

[35] Romero-Díez R., Rodríguez-Rojo S., Cocero M.J., Duarte C.M.M., Matias A.A., Bronze M.R. (2018). Phenolic Characterization of Aging Wine Lees: Correlation with Antioxidant Activities. Food Chem..

[36] Reis G.M., Faccin H., Viana C., da Rosa M.B., de Carvalho L.M. (2016). *Vitis vinifera* L. Cv Pinot Noir Pomace and Lees as Potential Sources of Bioactive Compounds. Int. J. Food Sci. Nutr..

[37] Ruskovska T., Maksimova V., Milenkovic D. (2020). Polyphenols in Human Nutrition: From the in Vitro Antioxidant Capacity to the Beneficial Effects on Cardiometabolic Health and Related Inter-Individual Variability-An Overview and Perspective. Br. J. Nutr..

[38] Pellegrini N., Serafini M., Colombi B., del Rio D., Salvatore S., Bianchi M., Brighenti F. (2003). Total Antioxidant Capacity of Plant Foods, Beverages and Oils Consumed in Italy Assessed by Three Different in Vitro Assays. J. Nutr..

[39] Ioele G., De Luca M., Oliverio F., Ragno G. (2009). Prediction of Photosensitivity of 1,4-Dihydropyridine Antihypertensives by Quantitative Structure-Property Relationship. Talanta.

